# The hallmarks of autism

**DOI:** 10.3389/fpsyt.2022.937163

**Published:** 2022-08-02

**Authors:** Bernard J. Crespi

**Affiliations:** Department of Biological Sciences, Simon Fraser University, Burnaby, BC, Canada

**Keywords:** autism, syndrome, Kanner, prototypes, diagnosis

## Abstract

I suggest that the current study of autism is problematic, due to: (1) its failure to pursue a medical model of disease causation, with protocols for differential diagnoses of causes; (2) a notable incidence of unrecognized false positive diagnoses in children; (3) the conceptual equating of autism with sets of traits that have been shown to be genetically and phenotypically unrelated to one another; and (4) the expansion of use of the terms “autism” and “autism traits” to psychiatric conditions that have no substantive etiological or symptomatic overlap with autism. These problems can be alleviated by, like Kanner, considering autism as a syndrome, a constellation of traits, conceptualized as differences rather than deficits, some set of which is found in each affected individual to some degree. The original, prototypical form of autism can be delineated based on the “hallmarks” of autism: a set of core traits, originally explicated by Kanner, that defines a relatively-homogeneous group, and that connects with the larger set of autism symptoms. The hallmarks of autism provide a touchstone for research that is unambiguous, historically continuous to the present, and linked with major theories for explaining the causes and symptoms of autism. Use of the hallmarks of autism does not impact recognition and treatment of individuals with DSM diagnosed autism, or individuals with the many disorders that involve social deficits. This perspective is compatible with the research domain criteria approach to studying autism, *via* analyses of autism's constituent traits and the differential diagnosis of its individual-specific causes.

## Introduction

The purpose of this article is to suggest that the current conceptualization and study of autism are highly problematic, with notable deleterious impacts on the efficacy of empirical research and the development of better clinical protocols and applications. A simple solution is proposed, based on ideas developed by Mottron (1, 2) and on what I call Kanner's “hallmarks” of autism.

I first describe the standard medical model of human disease, and show how it does not apply to the main, current psychiatric model for mental disorders in general and autism in particular. Second, I discuss salient findings on the genetic and phenotypic heterogeneity of autism, in the context of the history of its diagnostic criteria. This heterogeneity has led to conceptual expansion of the “autism spectrum” and “autism traits” such that they have become largely synonymous with social deficits and lose meaning as psychological-psychiatric constructs with any useful specificity. These changes have also apparently led to a substantial incidence of false positive autism diagnoses in childhood. Third, I provide specific suggestions for surmounting these problems, in the context of autism as originally described by Kanner (3), and centered on his “hallmarks” of autism, described below, as currently conceived.

## The standard medical model

The standard medical model of disease focuses on diagnosing what adaptive biological system has become maladaptive and dysfunctional in what way (4). Diseases are thus inextricably connected, conceptually and mechanistically, with specific adaptations. For example, lymphoma represents excessive lymphocyte replication, osteoporosis is defined as bone density that has become notably reduced, and type 1 diabetes is triggered by insufficient production of insulin. The causes of such diseases are discerned by studying the normal biological functioning of the adaptations, to understand how and why different dysfunctions occur and manifest in a disease and its symptoms. For each patient presenting initially with some set of symptoms, some process of implicit or explicit differential diagnosis is typically followed to determine the biological causes, which determine the optimal treatment.

In contrast to this model, mental disorders are considered predominantly in terms of symptomatic deficits in cognition, mood, and behavior, and the presence of some pattern or patterns of dysfunctional or distorted cognition, mood or behavior. Diagnostic procedures are used, in DSM or ICD frameworks, to determine what named disorder best fits a particular subject. Subjects are then provided some category of psychological or pharmacological treatment, based on their diagnosis. This approach is pragmatic in a societal framework but it is also becoming more and more limited, scientifically and clinically, as the genetic and biological bases of mental disorders have become better understood.

The primary difficulties with the psychiatric model of disease are two-fold. First, nominal disorders are commonly reified (considered as “real” when they are not, because the relevant adaptations and specific biological dysfunctions have not been defined or delineated), despite the fact that their descriptions and categories have changed, sometimes profoundly, every five, ten or fifteen years (5). Reification implies truth that does not exist, and promotes use of broad, formalized, and inflexible categories without questioning their scientific bases. It also encourages people to believe that diagnoses of autism by clinicians are not only biologically real (having biological coherence in terms of dysregulated adaptations) but also necessarily always correct, rather than representing hypotheses that may turn out to be false positives. Most generally, and in keeping with the standard medical model described above, mental disorders such as autism can more usefully be considered as “harmful dysfunctions” (6, 7), where “dysfunction” represents a scientific criterion that refers to specific mental traits that are not performing their evolved, adaptive functions, and “harmful” represents a cultural, value-based criterion determining whether or not a set of mental traits (a putative disorder) are considered as problematic for the individual or individuals concerned (6, 7). In the context of human neurodiversity, and subjective experiential wellbeing, many “autistic” individuals indeed consider their “disorder” to be nothing of the kind [e.g., (8)].

Second, because the differential diagnostic process aims at DSM or ICD diagnoses, it usually stops there. As such, psychiatrists, and other medical professionals, normally do not attempt to ascertain the biological causes of a person's psychiatric problems, by gathering data on known causes and correlates, aside from rare genetic risk factors. In this framework, the causes of autism can be depicted as tracings from genes, through development, to different levels of phenotypes. Every individual diagnosed with autism can be represented as expressing a different trajectory to a similar endpoint: some set of diagnostic traits. Most importantly, there have been virtually no attempts to develop efficient protocols for differential diagnosis of the biological causes of autism, to recover this trajectory as best possible. Presumably, the absence of such efforts stems in part from the known high heterogeneity of autism as regards symptom profiles, intelligence, genetic and environmental bases, and ultimately causes. How can such heterogeneity be addressed?

## The heterogeneity of autism

Autism was once considered to be a unitary disorder, with a single cause. Happé et al. (9) showed, using twin data, that the three main characteristics of autism as then conceived, (a) social impairment, (b) communication difficulties, and (c) repetitive and rigid behaviors and interests, were mainly independent of one another genetically and phenotypically. Autism was thus “fractionable” into these domains, and did not, by this evidence, exist as a clearly, coherent entity. Mandy and Skuse (10) similarly reported a lack of evidence for association of social with restricted-interests, repetitive behavior dimensions of autism, in a review of evidence available to date, and Robinson et al. (11) found notably low genetic and environmental correlations between these two domains. Comparable supporting results, at the genomic level for the first time, were recently reported by Warrier et al. (12), who found that genetic risk for a non-social autism-related trait (“systemizing”) was independent of genetic risk for social autism traits. Taken together, these studies suggest that current studies of autism often confound its social and non-social aspects. How can genomic architecture and causes be analyzed for a psychiatric construct that may not, as a unitary phenomenon, even exist?

The approach taken by most geneticists is to retain the term “autism” as the focus for their analyses, and to continue searching for “core” autism genes, perhaps also taking account of apparent heterogeneity in causes by seeking to identify autism “subtypes” (13), or by expanding analyses to include additional “neurodevelopmental disorders” (as a higher-level diagnostic category itself), like schizophrenia [e.g., (14)]. The degree to which autism subtypes exist as any sort of distinct “types,” and how they might be identified, remains an open question. A broader issue is that, given high levels of both genetic and phenotypic heterogeneity in the psychological traits found among people diagnosed with autism, what exactly GWAS studies of autism are measuring, and how their findings can ever be made useful for diagnoses, causal understanding or treatment. Ultimately, and as suggested originally by Rutter (15), what we may need is GWAS, and other analyses, of variation in each of the adaptive neurological and cognitive systems that may be altered in people diagnosed with autism. After all, we need to understand how adaptive systems actually develop and work before we can understand the many ways that they vary and can become problematic. And a key adaptive system, in autism as well as many other disorders including, especially, schizophrenia, is normally considered to be social cognition.

## Social deficits, autism, and schizophrenia

Autism-related social traits were reconceptualized in terms of cognitive and social impairments by Wing and Gould [see (5, 16)]; before that, description of autistic phenotypes could be traced to Kanner (3) who did not discuss social or cognitive deficits or impairments at all (5). As described by Evans (5), Wing and Gould acted from a desire to expand the pool of children who could be recognized as “autistic” and thereby helped by medical systems. From 1980 until now, characterization and measurement of social and cognitive deficits have dominated the diagnosis and study of autism. Throughout most of this period, an autism spectrum disorder could indeed be diagnosed based on social impairments alone (e.g., as PDD-NOS). The autism spectrum broadened in other ways, through the adoption of such concepts and metrics as the “broad autism phenotype,” the “Autism Quotient,” and use of the term “autism traits” to refer to social deficits (17, 18). The equating or conflating of autism with various forms of social and cognitive deficits has led, for example, to studies finding high levels of “autism traits,” or diagnoses of autism, by Autism Quotient scores or other metrics, among subjects with anorexia (19), suicide attempts (20), borderline personality (21), and schizophrenia spectrum disorders (22). Such findings are interpreted as indicating that each of these conditions overlaps with, and is often comorbid with, autism, and also includes so-called “autism traits.” Other studies, such as those that apply questionnaires to quantify “autistic features” among individuals with schizophrenia [e.g., (23, 24)], are based in Bleuler's century-old characterization of schizophrenia, and ignore the fact that Bleuler's view of “autistic” cognition was profoundly different, and in some ways opposite, to that described by Kanner (5, 25) and Crespi (26).

An alternative explanation for reports of “autism” or “autism traits” in non-autistic populations, from Rutter (15), is that “almost any mental disorder will impinge on social functioning to some degree or other.” The conceptual “explosion” of autism and “autism traits” to include social deficits has indeed apparently driven, in substantial part, the increases in autism diagnoses over time, and the decreases in effect sizes found among studies that compare “autistic” with “control” groups (1). It has also, as described below, essentially obliterated Kanner's view of autism.

Conflation of autism with social impairments is especially problematic given that social difficulties are common and pronounced in many children who are premorbid for schizophrenia (27, 28), (or with other disorders), but whose only option for diagnosis, during most of the periods of GWAS and CNV (copy-number variation) studies, has been the autism spectrum, including PDD-NOS. False positive diagnoses of schizophrenia premorbidity as autism spectrum, due in large part to the considered primacy of social-cognitive deficits in child psychiatry, may, by the views presented here, have systematically misled a generation of researchers, as detailed by Crespi et al. (27), Crespi and Crofts (28), and Crespi (29). Such conflation may also have resulted in the weak positive genetic correlation between schizophrenia and autism found in some studies (30), and the belief that reciprocal CNVs, which involve opposite deviations from typical average values for diverse neurological and anatomical traits, cause the same deviation as regards psychiatric diagnosis of autism (29). There is indeed no unambiguous or substantive neurological evidence for causal, etiological overlap of autism with schizophrenia (31), and overlap in “social deficits” (e.g., of “autism traits” with negative symptoms of schizophrenia) is irrelevant without data on their causes and biological bases.

If autism, then, is neither social and cognitive deficits, nor social deficits combined with restricted interests and repetitive behaviors, nor an overlapping facet of schizophrenia, what is it? I would suggest: what Kanner (3) said it is: a “syndrome.” In medicine and psychiatry, a “syndrome” can be conceptualized simply as a constellation of phenotypic traits and differences that shows some tendency to be found together in sets of individuals or that, when found together, causes particular sorts of problems. A syndrome may thus comprise a set of morphological, neurological, physiological, behavioral and developmental traits, each of which shows some level of difference from the age- and gender-typical average. Any given individual exhibits some degree of expression of each trait that, taken across them, is individual-specific. The traits that comprise a syndrome are thus discrete, but their levels of expression are continuous. Some sets of traits may tend to be found together, statistically, and one or more traits may be found at some level in all individuals considered to exhibit the syndrome. High expression of particular traits or sets of them may be indicative of specific psychological difficulties and expected benefits from particular forms of care and treatment. Van Os (32) described schizophrenia as exhibiting such a structure, and he referred to it as “salience syndrome.” The term “syndrome” as used here applies to idiopathic (cause-unknown) conditions, such as autism or schizophrenia, not to genetic syndromes, such as Fragile X syndrome or Down syndrome, which involve a known genetic cause for their particular sets of associated phenotypes. As applied here, recognition of the syndrome of autism is not linked to its causal mechanisms, which will vary notably from person to person.

Syndromes are characterized by a set of traits, and they exhibit four key properties. First, syndromes constitute multiple dimensions (their constituent phenotypes), each of which varies in degree of expression. As such, considering autism as a one-dimensional spectrum *per se*, such as along a line from low to high functioning or severity, is incompatible with their structure (33). This consideration means that the term autism “spectrum” may itself be misleading, because the term means unidimensionality of a singular construct between two points. Autism is, by contrast, multidimensional.

Second, syndromes are, or should be, made up of traits that are associated with adaptation in some way. As such, each trait characteristic of a syndrome is expected to be causally connected with one or more neurological structures or functions. Differences from typical or average function can thus be analyzed in the context of the standard medical model and the research domain criteria approaches. As such, the components of a syndrome are “real,” in the sense that they represent alterations to, or variation in, evolved adaptations (e. g., specific aspects of human cognition with neurological bases) that have become more or less maladapted, and might also be considered as harmful or problematic. Most importantly, such components need not be based on, or defined by, deficits *per se*, just differences. Indeed, social and communicative deficits as measured among individuals with autism may well represent secondary effects of the primary differences described by Kanner.

**Figure 1:**
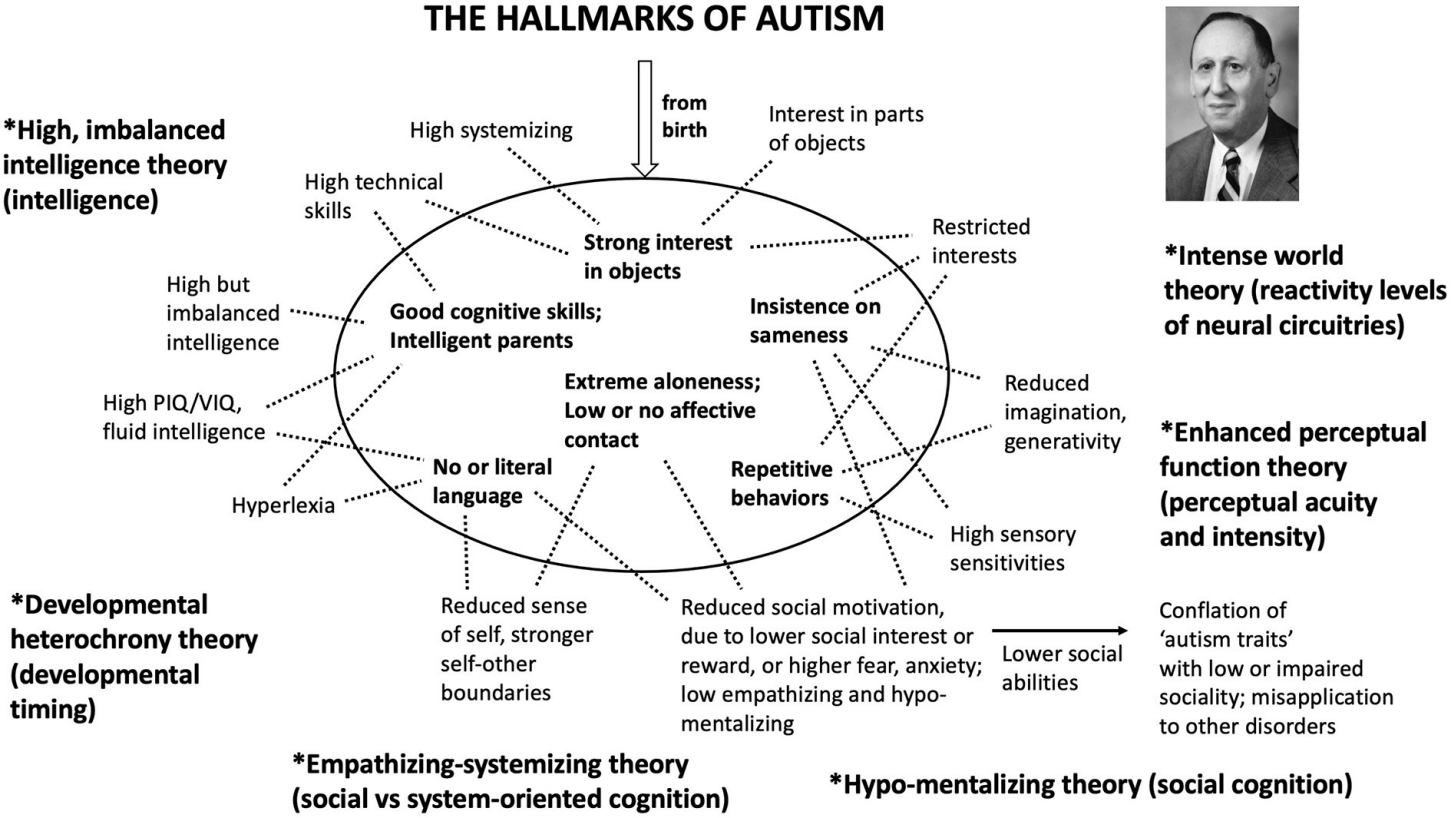
The six main “hallmarks of autism” described by Kanner in his 1943 paper. These hallmarks represent the core phenotypes that Kanner used to define and describe the syndrome of autism. Each of them has since been connected (here, by the dotted lines) with additional traits that are associated with autism. They are also linked with the main theories set forth for understanding autism (here, in boldface); these theories include systemizing and empathizing (34), enhanced perceptual function and veridical mapping (35, 36), neuronal hyper-excitability and plasticity (37), and high but imbalanced intelligence, hyperdeveloped patternistic cognition, developmental heterochrony, and hypo-developed mentalizing (38–40). Each theory is followed in parentheses by the adaptation(s) that, by the theory, are altered in autism. These theories show evidence of strong connections with one another, especially as regards intelligence with high perceptual function and neural reactivity, and low empathizing with hypo-mentalism; relatively or absolutely enhanced non-social cognitive abilities and interests are also prominent in all of them.

Third, the boundaries of syndromes, in terms of the specific collection of traits that comprises them, can be “fuzzy”: some such traits are found in all or most affected individuals, but other traits are less common. As a result, delineation of a set of traits characteristic of a syndrome is necessarily arbitrary to some degree, once one moves beyond any traits that are considered necessary for the syndrome to be recognized. Figure 1 thus depicts one delineation of “hallmarks” for autism, drawn directly from Kanner. There could be others, derived empirically (2), and equally or more useful in terms of guiding research and, ultimately, helping individuals.

Fourth, syndromes naturally promote protocols for finding individual-specific etiology, because they dictate measurement across a suite of syndrome-associated traits. Individual etiology is real, and its diagnoses lead to understanding of causes that may be more or less general, or specific. Such precision diagnostic medicine can lead directly to personalized optimization of therapies.

Kanner's description of autism as a syndrome centers on a set of traits that were characteristic across the eleven individuals who he originally studied. These traits, extracted from his 1943 article and depicted in Figure 1, represent his main “hallmarks” of autism: the primary distinguishing features that he used to recognize it as a psychiatric entity in the first place. Each of Kanner's hallmarks can be connected with one or more specific autism-associated traits from more-recent studies, and, taken together, these can all be linked with core theories for understanding autism (Figure 1). In principle, Kanner's hallmarks should also be underlain by differences, between individuals with and without autism, in neurological traits that jointly subserve human abilities in the domains that are shared by these theories, especially as regards enhanced motivation toward, and recognition and processing of, non-social information as found in patterns, systems, and integrated structures (34, 39, 41, 42).

Of his six hallmarks, Kanner considered the construct of “aloneness” as being characteristic, most broadly, of autism as he conceived it. His hallmarks remain useful for research insights, in that, for example, “aloneness,” “interest in objects” and “insistence on sameness” are all central aspects of autism that have been largely ignored as regards their neurological and genetically-based causes. Kanner's collection of autism-diagnostic traits also overlaps substantially with the of Asperger (43), excepting Asperger's increased focus on individuals with relatively developed language abilities and less-developed repetitive behavior. Perhaps most importantly, by Kanner's hallmarks of autism, social and communicative deficits may represent secondary effects of autistic development, and not primary, causal, or usefully diagnostic manifestations of the condition itself.

Considering autism as a syndrome, as Kanner did, need have little or no impact upon current diagnostic criteria, which serve a variety of goals in communication and flagging of individuals who may benefit from support. However, as regards the conduct of research, a syndromic view of autism, and differential diagnosis of autism's diverse manifestations and causes, are likely to be considerably more productive than current alternatives. In particular, focusing research studies on individuals with “prototypical” autism, and on the collecting of data to better-define autism prototypes (1, 2), as well as autism defined by criteria compatible with Kanner's hallmarks, will help to better ensure that autism researchers are all studying a closely-similar condition, and will help to connect the “harmful dysfunctions” involved in autism with the relevant underlying adaptations. As such, clinical and research strategies for scientific studies of autism become partially dissociated, with clinical work focusing on individualized diagnoses in the syndrome context as well as the DSM or ICD frameworks, research work characterizing and quantifying heterogeneity in study populations as an integral and essential part of every study, and treatments following from protocols designed to indicate more or less individualized causes and correlates. Such a framework will, at very least, help to prevent further untoward and misleading expansion of the concept of autism away from its well-founded roots.

## Data Availability Statement

No datasets were generated for this study.

## Author contributions

The author confirms being the sole contributor of this work and has approved it for publication.

## Funding

Funded by the Natural Sciences and Engineering Research Council of Canada (Discovery Grant 2019-04208).

## Conflict of interest

The author declares that the research was conducted in the absence of any commercial or financial relationships that could be construed as a potential conflict of interest.

## Publisher's note

All claims expressed in this article are solely those of the author and do not necessarily represent those of their affiliated organizations, or those of the publisher, the editors and the reviewers. Any product that may be evaluated in this article, or claim that may be made by its manufacturer, is not guaranteed or endorsed by the publisher.
